# Treatment strategy introducing immunosuppressive drugs with glucocorticoids ab initio or very early in giant cell arteritis: A multicenter retrospective controlled study

**DOI:** 10.1016/j.jtauto.2020.100072

**Published:** 2020-11-28

**Authors:** Luca Quartuccio, Miriam Isola, Dario Bruno, Elena Treppo, Laura Gigante, Francesca Angelotti, Riccardo Capecchi, Gianfranco Vitiello, Elena Cavallaro, Antonio Tavoni, Silvia Laura Bosello, Daniele Cammelli, Salvatore De Vita, Elisa Gremese

**Affiliations:** a Department of Medicine, Rheumatology Clinic, Udine Academic Hospital “Santa Maria Della Misericordia”, Udine, Italy; b Department of Medicine, Institute of Statistics, University of Udine, Udine, Italy; c Rheumatology Unit, Catholic University of the Sacred Heart, Rome, Italy; d Department of Internal Medicine, Clinic of Immunology, Pisa, Italy; e University of Florence, Florence, Italy; f Division of Rheumatology, Fondazione Policlinico Universitario A. Gemelli-IRCCS, Rome, Italy

**Keywords:** Giant cell arteritis, Temporal arteritis, Treatment, Glucocorticoids, Methotrexate, Tocilizumab, Adverse events

## Abstract

**Objective:**

Glucocorticoids (GC) are associated with side effects in giant cell arteritis (GCA). Immunosuppressive therapies (ITs) have given conflicting results in GCA, regarding GC sparing effect. Primary endpoint is to evaluate whether very early introduction of ITs in GCA minimize the rate of GC-induced adverse events, in terms of infections, new onset systemic arterial hypertension, GC-induced diabetes and osteoporotic fractures.

**Methods:**

A multicenter retrospective case-control study included 165 patients. One group included 114 patients who were treated with at least one IT given at diagnosis or within 3 months from the start of GC. A second group included 51 GCA who received only GC or an IT more than 3 months later.

**Results:**

The most frequently used ITs were: methotrexate (138 patients), cyclophosphamide (48 patients) and tocilizumab (27 patients). No difference was observed as concerns the follow-up time between groups [48.5 (IQR 26–72) vs 40 (IQR 24–69), p ​= ​0.3)]. The first group showed a significantly lower incidence of steroid-induced diabetes (8/114, 7% vs 12/51, 23.5%; p ​= ​0.003) and no differences for the rate of infections (p ​= ​0.64). The group was also exposed to lower doses of GC at first (p ​< ​0.0001) and third (p ​< ​0.0001, rank-sum test) month. Forty-four patients in the first group (38.6%) compared with 34 in the second one (66.7%) experienced at least one relapse (p ​= ​0.001).

**Conclusion:**

Very early introduction of IT in GCA lowered the incidence of steroid-induced diabetes, possibly due to the lower doses of GC in the first three months. Relapse rate was even lower.

## Highlights

• Giant cell arteritis is still a glucocorticoid-dependent disease.
• Very early introduction of immunosuppressive agents minimizes the use of glucocorticoids.
• Incidence of diabetes could be decreased by this strategy.
• Safety in the elderly seems to be guaranteed.

## 1 Introduction

Giant cell arteritis (GCA) is the most common form of primary vasculitis [[1], [2], [3]]. It is a granulomatous inflammation of medium to large-sized vessels, mainly affecting cranial branches of arteries originating from the aortic arch, including the temporal arteries, with potentially disabling complications. Large artery complications are also increasingly well recognized [1,2,4]. Glucocorticoids (GC) remain the mainstay of treatment of GCA and guidelines usually recommend an initial prednisone dosage of 40–60 ​mg/day gradually tapered over a period of 1–2 years [5]. This strategy is usually effective in controlling the disease and preventing progressive blindness. However, relapses occur in up to 50% of patients when GC are tapered and prolonged courses of GC are associated with serious side effects, which greatly affect the outcome [6]. Thus, several studies have been conducted on the effectiveness of a GC-sparing immunosuppressive therapy (IT), yielding conflicting results. To date, three randomized controlled trials have evaluated the use of methotrexate in patients with newly diagnosed GCA, with two of them showing no steroid-sparing benefit [[7], [8], [9]]. A subsequent meta-analysis showed a modest benefit of methotrexate, with fewer relapses and lower GC exposure in patients on methotrexate [10]. Uncontrolled retrospective case reports and case series also reported the beneficial effects of cyclophosphamide, although burdened with possible serious adverse events (AEs) [[11], [12], [13], [14]]. Tocilizumab, a humanized monoclonal antibody directed against the IL-6 receptor, has been licensed for GCA, based on the results of a phase 2 trial and a large, multicenter, phase 3 randomized controlled trial [15,16]. Nevertheless, tocilizumab may have some limitations since it is an expensive drug, with some contraindications. On the other hand, still limited information is available regarding long-term outcome and steroid sparing effect of other ITs in GCA, while they may be of value as further options to improve the outcome of GCA patients.

## 2 Materials and methods

We retrospectively enrolled 165 patients with follow up details of at least one year from the departments of internal medicine and rheumatology at four tertiary hospitals located in Udine, Roma, Pisa and Florence. All patients were diagnosed with GCA between 2007 and 2016 based on the presence of at least 3 criteria of the American College of Rheumatology (ACR) [17] or demonstration of large vessel involvement on imaging. For the purpose of this study, we enrolled consecutive patients with GCA who received an adjunctive IT given very early after GCA diagnosis, i.e. ab initio or within 3 months from the start of GC (GCA-IT). These groups were compared with consecutive patients with GCA who never received an adjunctive IT or IT was given later than 3 months after GCA diagnosis (GCA-control). The data were collected in a retrospective manner from our institutions’ electronic medical records. The following were included in a standardized form: demographics, clinical manifestations and laboratory tests [including C-Reactive Protein (CRP), erythrocyte sedimentation rate (ESR)] at diagnosis and lastly follow up visit and temporal artery biopsy status when available. We also retrieved the dose of GC at treatment initiation and at months 3, 6, 12, 24 and 48, if ongoing, the number of disease relapse events, as well as the occurrence of the most commonly reported complications of GC, i.e. infectious events, new onset or deterioration of systemic arterial hypertension, steroid-induced diabetes (new or worsening of a pre-existing type 2 diabetes) and new fragility fractures. Remission was defined as the absence of symptoms or signs, along with ESR <40 ​mm/h or CRP <10 ​mg/l. Relapse was defined in the presence of either one of the signs and symptoms that newly appeared or worsened after achieving clinical remission. Given the retrospective nature of the study, no medical therapy protocol was adopted. The choice of IT was made by the treating physician according to his/her judgement of disease activity and patient comorbidity. Similarly, the initial GC dosage, as well as lower subsequent dosages, were based on the physician’s decision. Most patients received more than one IT during the follow-up, as this was modified in case of insufficient response or intolerance. The decision to give an immunosuppressant along with steroid therapy was made regardless of the severity of manifestations or presence of pre-existing comorbidities. Usually, the treatment strategy included methotrexate as first therapeutic choice, except for contraindications or very high disease activity. In the latter case, it was preferred to use cyclophosphamide (intravenous or orally) or tocilizumab intravenously or subcutaneously, when available. Informed consent to use clinical data was obtained from all individual participants included in the study. The study was conducted in accordance with the ethical principles of the Helsinki Declaration (WMA, 2013).

### 2.1 Statistical analysis

Descriptive statistics summarized data using the mean and standard deviation or median and interquartile range (IQR), as appropriate based on the variable distribution, or even frequencies for dichotomic variables; consequently, comparisons between GCA-IT and GCA-control groups were made by parametric (t-test for two independent samples) or no parametric tests (Mann-Whitney test) for continuous variable, while chi square tests for dichotomic variables. The log rank test was used to test the null hypothesis that there was no difference between GCA-IT and GCA-control groups in the probability of relapse as event, at any time point.

## 3 Results

### 3.1 Demographic and clinical features at onset

Overall, methotrexate up to 20 ​mg/week was employed in 138/165 (83.6%) patients, cyclophosphamide given monthly at the dose of 15 ​mg/kg intravenously adjusted for age and renal function, or given orally at the dose of 1.5–2 ​mg/kg/day for three months in 48/165 (29%) patients and tocilizumab either intravenously at the dose of 8 ​mg/kg per month or 162 ​mg weekly subcutaneously in 27/165 (16.4%) patients. GCA-IT group comprised 114 patients, while 51 patients belonged to GCA-control group. Characteristics of the two groups are reported in Table 1. Notably, there were no differences regarding sex distribution (p ​= ​0.134), while GCA-IT group patients were significantly younger than GCA-control patients (p ​= ​0.009, two-sample Wilcoxon rank-sum test-Mann-Whitney test), even if the mean age of each group was over 65 years. At least three classification criteria were satisfied by 81/114 vs 48/51 patients, respectively in GCA-IT and GCA-control group (p ​= ​0.002). No difference was noticed in the number of patients who had undergone temporal artery biopsy (96/114 vs 45/51, p ​= ​0.195), as well as in the rate of positive results from biopsy (40/96 vs 22/45, p ​= ​0.421). Yet, at onset, statistically significant differences between groups were recorded in the rate of temporal artery abnormalities (48/114 vs 30/51, p ​= ​0.047), and masticatory claudication (40/113 vs 28/51, p ​= ​0.019), but not headache, visual impairment, and extracranial manifestations, including polymyalgic symptoms, fever or low-grade fever. However, both cranial and extracranial ischemic complications appeared more frequent in the first group (Table 1). Start prednisone dosage was not statistically different between groups (p ​= ​0.066). Treatments employed in the GCA-IT group are described in Table 2. Time of observation was not statistically different between GCA-IT and GCA-control groups (p ​= ​0.304). Pre-existing comorbidity did not appear different between groups (table S1).

**Table 1:** Demographic, clinical, laboratory, instrumental and histological features of the GCA-IT and GCA-control groups.

variable	GCA-IT (N ​= ​114)	GC-control (N ​= ​51)	P value
Age at diagnosis, mean (SD)	68.9 (7.7)	72.3 (7.1)	0.005
Female, n (%)	84 (73.7)	43 (84.3)	0.134
1990 ACR Classification criteria, median (IQR)	3 (2–4)	4 (3–5)	0.005
Headache, n (%)	79 (69.3)	42 (82.3)	0.080
Jaw claudication, n (%)	40 (35.4)	28 (54.9)	0.019
Temporal artery abnormalities	48/114 (42.1)	30/51 (58.8)	0.047
PMR, n (%)	56 (49.6)	28 (54.9)	0.526
Fever, n (%)	50 (44.2)	23 (45.1)	0.919
Visual impairment, n (%)	22 (19.5)	16 (31.4)	0.094
Extracranial clinical involvement, n (%)	68 (59.6)	26 (51)	0.299
Ischemic complications (cranial and/or extracranial)	247,114 (21.1)	5/46 (9.8)	0.079
ESR, mm/h, mean (SD)	74 (30.6)	81.6 (25.4)	0.135
CRP, mg/l, median (IQR)	48 (14.3–96.1)	64.5 (37.25–105.25)	0.033
Positive PET-CT, n/N (%)	46/53 (86.8)	12/16 (75)	0.259
Positive temporal artery biopsy, n/N (%)	40/96 (41.7)	22/45 (48.9)	0.421
Follow-up, months, median (IQR)	48.5 (26–72)	40 (24–69)	0.304

Legend: SD, standard deviation; PMR, polymyalgia rheumatica; ESR, erythrocyte sedimentation rate; CRP, C-Reactive Protein; PET-CT, Positron emission tomography–computed tomography; CTA, computed tomography angiography; MRA, magnetic resonance angiography; IQR, interquartile range 25%–75%.

**Table 2:** Treatments employed in the two groups and outcomes.

Variable	GCA-IT (N ​= ​114)	GC-control (N ​= ​51)^a^
GC alone for the whole follow-up^a^, n (%)	0	19/51 (37.2)
Methotrexate, n (%)	105 (92.1)	33 (64.7)
Cyclophosphamide, n (%)	39 (34.2)	9 (17.6)
Tocilizumab, n (%)	20 (17.5)	7 (13.7)
Prednisone at onset, median (IQR)	50 (40–62.5)	50 (50–62.5)
Prednisone at the end of first month, median (IQR)	36.25 (20–50)^b^	50 (50–62.5)^b^
Prednisone at the end of third month, median (IQR)	12.5 (6.25–25)^b^	25 (12.5–37.5)^b^
Prednisone at the end of sixth month, median (IQR)	6.25 (5–10)	6.25 (5–10)
Prednisone at the end of twelfth month, median (IQR)	5 (3.75–5)	5 (5–6.25)
Prednisone at the end of twenty-fourth month, median (IQR)#	5 (0–5)	2.5 (0–5)
Prednisone at the end of forty-eighth month, median (IQR)§	1.75 (0–5)	0 (0–5)
Diabetes (%)	8/114 (7)°	12/51 (23.5)°
Infections (%)	19/114 (16.7)	10/51 (19.6)
Hypertension (%)	11/114 (9.6)	6/51 (11.7)
Fragility fractures (%)	15/114 (13.1)	4/51 (7.8)

a GCA-control group could be treated with IT after at least of three months of GC alone therapy.

b p value ​< ​0.0001; °p value ​< ​0.01; #data available in 150 (103 in GCA-IT, 47 in GCA-control) patients; §data available in 117 (78 in GCA-IT, 39 in GCA-control) patients.

### 3.2 Outcomes

#### 3.2.1 Primary outcome: GC-related complications

During the whole follow-up, the number of infectious complications was not statistically different between groups (19/114 vs 10/51, p ​= ​0.646, Pearson chi square test), even for those requiring hospitalization (5/19 vs 1/10, p ​= ​0.583, Pearson chi square test). Importantly, the rate of steroid-induced diabetes was significantly lower in the GCA-IT group (8/114 vs 12/51, p ​= ​0.003). The number of patients with new onset or worsening arterial systemic hypertension was not different (11/114 vs 6/51, p ​= ​0.680, Pearson chi square test), as well as for the rate of new fragility fractures (15/114 vs 4/51, p ​= ​0.323, Pearson chi square test).

#### 3.2.2 Secondary outcomes: steroid sparing, relapse rate, adverse events

The ongoing prednisone dose at the end of the first month, as well as at the end of the third month, was significantly lower in the GCA-IT group [36.25 ​mg/day (IQR 20–50) vs 50 ​mg/day (IQR 50–62.5); 12.5 ​mg/day (IQR 6.25–25) vs 25 ​mg/day (IQR 12.5–37.5), respectively, p ​< ​0.0001, two-sample Wilcoxon rank-sum test-Mann-Whitney test in both analyses], while no differences were observed in the prednisone daily dose between groups at the end of the sixth [6.25 ​mg/day (IQR 5–10) vs 6.25 ​mg/day (IQR 5–10), p ​= ​0.401, two-sample Wilcoxon rank-sum test-Mann-Whitney test] and twelve month [5 ​mg/day, IQR (5–6.25) vs 5 (IQR 3.75–5), p ​= ​0.069, two-sample Wilcoxon rank-sum test-Mann-Whitney test].

The number of patients showing at least one relapse was significantly lower in GCA-IT group than the GCA-control group (44/114 vs 34/51, p ​= ​0.001), while the number of patients with more than one relapse was not different among them (9/44 vs 8/34, p ​= ​0.960, Pearson chi square test). There was no difference in terms of time to first relapse between the two groups [360 days (150–1081) vs 180 days (90–510), p ​= ​0.144, log-rank test). Importantly, time (months) to reach 5 ​mg/day of prednisone was significantly lower in the GCA-IT group [6 (4–12) vs 9 (6–12), p ​= ​0.016)].

The total number of adverse events was not significantly different between the two groups (51/114 vs 29/51, p ​= ​0.203). Among them, the number of patients with more than one adverse event was again not statistically different (8/51 vs 7/29, p ​= ​0.527). Adverse events other than infections secondary to the IT in the GCA-IT group are reported in Table 3.

**Table 3:** Adverse events other than infections ascribed to immunosuppressors (as clinical judgement) in GCA-IT group.

Therapy	N° patients	Adverse events	Grade	Therapy modification	Outcome
Methotrexate, n ​= ​35	1	Hypertransaminasaemia	1	None	Resolved
	1	Hypertransaminasaemia	1	Dose reduction	Resolved
	7	Hypertransaminasaemia	2	Stop	Resolved
	3	Nausea	1	None	Resolved
	4	Nausea	1	Dose reduction	Resolved
	5	Nausea	1	Stop	Resolved
	1	Neutropenia	2	Dose reduction	Resolved
	1	Malaise	1	None	Resolved
	1	Malaise	1	Dose reduction	Resolved
	3	Malaise	1	Stop	Resolved
	1	Oral mucositis	2	Stop	Resolved
	1	Oral mucositis	1	Dose reduction	Resolved
	1	Stomatitis	1	Stop	Resolved
	2	Anaemia	2	Stop	Resolved
	1	Alopecia	1	Stop	Resolved
	1	Skin rash	1	Stop	Resolved
	1	Diarrhoea	1	Stop	Resolved
Cyclophosphamide, n ​= ​8	1	Hypertransaminasaemia	2	Stop	Resolved
	2	Haemorrhagic cystitis	1	None	Resolved
	1	Hypogammaglobulinemia	1	None	Resolved
	1	Thrombocytopenia	1	Dose reduction	Resolved
	1	Laryngeal cancer	3	Stop	Persisted
	1	Oral mycosis	1	Stop	Resolved
	1	Abdominal pain	1	Stop	Resolved
Tocilizumab, n ​= ​6	2	Thrombocytopenia	1	Dose reduction	Resolved
	1	Urticarial rash	1	Dose reduction	Resolved
	1	Leukopenia and thrombocytopenia	2	Stop	Resolved
	1	Thrombocytopenia and hypogammaglobulinemia	1	None	Resolved
	1	Neutropenia	1	None	Resolved

## 4 Discussion

To the best of our knowledge, the current study constitutes the largest case-control study of GCA comparing a strategy with an upfront use of steroid sparing ITs from the beginning or very early from the diagnosis with the standard of care, i.e. GC alone. The time of observation of more than three years in the majority of the patients was long enough to evaluate critical clinical outcome in this disease. Our proposed approach was effective in significantly decreasing the rate of steroid-induced diabetes, while not increasing the rate of infections, including the most serious ones requiring hospitalization, which was recorded at low rate in both groups, if compared to a previous work (18). Notably, the methotrexate arm of Mahr’s study reported an infection rate very close to that documented in the GCA-control arm of our study, i.e. 20% (10), where the introduction of IT was delayed. This important result was likely related to the steroid sparing effect that was clearly seen in our study at the end of the first and the third month, when the ongoing GC dose was significantly lower in GCA-IT group than in controls. Recently, Matthew J. Koster et al. reported a retrospective analysis in 83 GCA patients, treated with methotrexate and compared with a control group, which was managed only with GC. The median time to start methotrexate was 39 weeks, i.e. more than 9 months. The rate of relapses significantly decreased in the MTX group, but the GC reduction was not different between the two groups, while the cumulative steroid dose was significantly higher in the methotrexate group than in the control group [19]. This was justified by the fact that patients taking methotrexate showed a more severe disease before methotrexate initiation, 24 patients (29%) had not yet experienced a relapse, while, at index date, 59 (71%) patients receiving only GC had not yet experienced a relapse. Thus, in this clinical scenario and in the absence of biomarkers of severity for GCA at the time of diagnosis, only the very early introduction of IT may produce a clinically significant change in the outcome in GCA, regardless of the severity of the disease. The absence of difference between the groups regarding the rate of fragility fractures may be due to the large use of bisphosphonates along with calcium and vitamin D supplementation as bone protection prophylactic treatment, usually employed in this setting. Notably, despite the more rapid deescalating steroid schedule in GCA-IT group, the relapses remained significantly lower in that group than in GCA-control group, and it was even lower than that reported by a recent meta-analysis [20], thus underlying the early synergic immunosuppressive effect of IT with GC, starting from the first month, when the dosage of immunosuppressive therapies is appropriate. In fact, while the rapid effect of cyclophosphamide or tocilizumab is known, for methotrexate, reaching the maximum well-tolerated dosage, i.e. up to 20 ​mg/week within the first two weeks is critical. Mahr et al. performed a meta-analysis based on data from the three randomized controlled trials [[7], [8], [9]] which evaluated the use of methotrexate with conflicting results. The meta-analysis compared two arms of treatment, the first one treated with methotrexate and prednisolone, the second one including 77 patients treated with placebo and prednisolone [10]. Moreover, if compared to the placebo arm, methotrexate produced a significantly better outcome only after 24 weeks [10]. Since patients in the methotrexate group were not more severe than those in the placebo group, it may be hypothesized that the dosage of methotrexate could be really critical. In fact, in the study by Mahr et al. methotrexate was administered at a maximum dosage ranging between 7.5 ​mg/week and 15 ​mg/week, except in 1 patient whose dosage of methotrexate was gradually increased to 17.5 ​mg/week [10]. Thus, this analysis further supports the effectiveness of methotrexate at higher dosage in reducing relapses if compared with GC alone. Mahr et al. have already demonstrated in their meta-analysis that treatment with methotrexate decreased the cumulative exposure to GC [10], while they did not recommend a more rapid steroid tapering regimen, due to the possible delayed effect of adjunctive IT [10], which is expected at those lower dosages.

With regard to cyclophosphamide, current evidence on its role in GCA is scarce and limited to retrospective studies and case reports, including a retrospective study conducted by our group [11], where it appears to be effective in reducing steroid use within the first 6 months. In our study, it appears that cyclophosphamide was chosen for patients showing a more aggressive disease, in particular for those with ischemic complications and/or extracranial, large vessel involvement (Table 1). Interestingly, large vessel involvement at diagnosis has been recently associated with reduced survival in GCA [21], and, therefore, it may justify the choice of a deeper immunosuppression. Conversely, efficacy of tocilizumab in reducing GC exposure was assessed and clearly demonstrated by several studies, and above all by the GiACTA trial [16], which led to the authorization of its commercial use in GCA. Nevertheless, no study, including Mahr’s meta-analysis [10] before ours, has so far demonstrated a reduction in the rate of adverse events secondary to GC therapy. Globally, all other series were characterized by a significantly lower use of IT [6,10,18,22,23]. This outcome probably requires a long-term follow-up and, more importantly, not only new drugs, but also a new treatment algorithm, including early (within the first three month) introduction of an IT and a treat-to-target strategy. Nevertheless, some patients experienced adverse events, usually grade 1 or 2, secondary to IT, which, however, led to therapy withdrawal only in 14/114 (12.3%). Anyway, in all these cases an alternative IT was successfully started, and well tolerated. Our study has several limitations. First, the retrospective design limited the completeness of the retrieved data, especially the follow-up data. In addition, the treatment was not standardized and both initial dose and tapering regimens were at the discretion of the treating physician, although all the recruiting centres usually adopted a similar GC schedule in order to minimize the GC exposure in their own patients. Randomized controlled trial or observational prospective cohort study with the aim to determine long-term outcome using uniform treatment protocol would be needed and it has been recently claimed for methotrexate [24]. Further, patients undergoing IT were slightly younger from a statistical point of view, though it was not clinically relevant in our opinion. Otherwise, younger age may be a safety reason for the clinician to introduce IT in GCA early. Indeed, the 1990 ACR classification criteria for GCA were lesser satisfied by patients belonging to GCA-IT group than controls. In the former, typical specific cranial involvement and a positive temporal artery biopsy were less frequent, whereas they tended to show predominantly extra-cranial manifestations. This data definitely reflects the characteristics of real-life patients with GCA and underlines the limited sensitivity of the current classification criteria, especially in those patients with large vessel involvement. Similarly, the GiACTA trial enrolled 46% patients diagnosed with GCA based on imaging [16]. Provisional new classification criteria for GCA appear to have definitely incorporated this concept [25].

## 5 Conclusion

This study suggests that a very early introduction of IT, i.e. within the third month from diagnosis, could be a valid therapeutic strategy, especially in a population in which GC use must be minimized [26]. This strategy, in our study, was definitely effective in decreasing GC-related adverse effects in the long term, by lowering the exposure to GC early and reducing the risk of relapse. This is the main objective of treatment in GCA, which is the most frequent primary vasculitis in the adults, and it often affects elderly patients [27]. The choice of the type of immunosuppressant should be individualized based on the severity of the manifestations, comorbidity, contraindications, and, finally, on the available resources [28]. At present, comorbidity such as hypertension, venous thrombosis or diabetes at diagnosis, or systemic or local signs of much more inflamed disease (i.e. fever, anaemia or histological signs of more active disease) may predict relapse in GCA [18,29,30], which is much more frequent in the first year of treatment [20]. Indeed, no valid biomarkers have been found to predict which patients are at most risk for developing GC resistance in GCA [31]. Further prospective studies are needed to confirm these findings and try to personalize treatment strategy in each patient.

## Funding

“This research received no external funding”.

## Credit author statement

Conceptualization, Luca Quartuccio; Data curation, Elena Cavallaro, Francesca Angelotti, Laura Gigante, Dario Bruno, Riccardo Capecchi and Gianfranco Vitiello, Silvia Laura Bosello; Formal analysis, Luca Quartuccio, Miriam Isola; Investigation, Luca Quartuccio, Elena Cavallaro, Silvia Laura Bosello, Daniele Cammelli, Antonio Tavoni, Elisa Gremese; Methodology, Luca Quartuccio, Miriam Isola, Elisa Gremese, Salvatore De Vita; Project administration, Luca Quartuccio and Elena Cavallaro; Software, Luca Quartuccio and Elena Cavallaro; Supervision, Luca Quartuccio, Daniele Cammelli, Elisa Gremese, Antonio Tavoni, Salvatore De Vita; Validation, Luca Quartuccio, Elisa Gremese; Writing – original draft, Luca Quartuccio; Writing – review & editing, Luca Quartuccio, Elena Cavallaro, Francesca Angelotti, Riccardo Capecchi, Gianfranco Vitiello, Laura Gigante, Dario Bruno, Silvia Laura Bosello, Elisa Gremese, Daniele Cammelli, Antonio Tavoni, Salvatore De Vita.

## Declaration of competing interest

The authors declare that they have no known competing financial interests or personal relationships that could have appeared to influence the work reported in this paper.
