# Supplementary material for: Treatment strategy introducing immunosuppressive drugs with glucocorticoids ab initio or very early in giant cell arteritis: A multicenter retrospective controlled study
Source: J Transl Autoimmun. 2020 Nov 28;3:100072. doi: 10.1016/j.jtauto.2020.100072 (PMC7718148; doi:10.1016/j.jtauto.2020.100072)
Supplement: Multimedia component 1 [file mmc1.docx]

**Supplemental file.**

**Table S1**. Pre-existing co-morbidity

| **variable** | **GCA-IT (N=82)** | **GC-control (N=50)** | **P value** |
| --- | --- | --- | --- |
| Pre-existing type 2 diabetes, n (%) | 10 (12.2) | 6 (12) | 0.973 |
| Arterial systemic hypertension, n (%) | 42 (51.2) | 30 (60) | 0.326 |
| Osteoporosis, n (%) | 15 (18.3) | 5 (10) | 0.197 |
| Ischemic heart disease, n (%) | 2 (2.4) | 0 | 0.266 |
| Non ischemic heart disease, n (%) | 6 (7.3) | 1 (2) |  |
| History of cancer, n (%) | 4 (8) | 15 (18.3) | 0.102 |
| Other chronic comorbidity*, n (%) | 32 (39) | 12 (24) | 0.076 |

*including chronic renal insufficiency, thyroid disease, glaucoma, chronic respiratory diseases, peripheral arterial occlusive disease, liver steatosis, cerebrovascular disease, parkinson disease, gastroesophageal rflux disease.
